# Translation into Turkish of: “Changes to publication requirements made at the XVIII International Botanical Congress in Melbourne – what does e-publication mean for you?”. Translated by Ali A. Dönmez, Yusuf Menemen and Zübeyde Uğurlu. “Onsekizinci (XVIII) Uluslararası Botanik Kongresi’nde (Melbourne) Yayın ile I˙lgili Gereklilikler Konusunda Yapılan Değişiklikler; e-yayın size ne ifade ediyor”

**DOI:** 10.3897/phytokeys.7.2320

**Published:** 2011-11-29

**Authors:** Sandra Knapp, John McNeill, Nicholas J. Turland

**Affiliations:** 1 Department of Botany, The Natural History Museum, Cromwell Road, London SW7 5BD, UK The Natural History Museum London United Kingdom; 2 Royal Botanic Garden, Edinburgh, 20A Inverleith Row, Edinburgh EH3 5LR, UK Royal Botanic Garden Edinburgh United Kingdom; 3 Missouri Botanical Garden, PO Box 299, St Louis, MO 63166-0299, USA Missouri Botanical Garden St. Louis United States of America

## Abstract

Uluslararası Botanik Adlandırma Kuralları’nda yapılan değişikliklere, her altı yılda bir gerçekleştirilen ve Uluslararası Botanik Kongresi (IBC) ile beraber yapılan Adlandırma Seksiyonlarında karar verilmektedir. On sekizinci (XVIII.) Uluslararası Botanik Kongresi, Avustralya’nın Melborn şehrinde yapılmıştır. Adlandırma Seksiyonu 18-22 Temmuz 2011 tarihleri arasında toplanmış olup, kararlar 30 Temmuzdaki genel kurul toplantısında kongre tarafından kabul edilmiştir. Bu toplantıda yeni isimlerin yayınlanması ile ilgili olarak *Yasa*’da bazı önemli değişiklikler yapılmıştır. Bu değişikliklerden iki tanesi *Melborn Yasası* basılmadan bir süre önce, 1 Ocak 2012 tarihinden itibaren geçerli olacaktır. 1- Elektronik ortamda Uluslararası Standart Seri Numarası (ISSN) ya da Uluslararası Standart Kitap Numarası (ISBN)’na sahip Taşınabilir Belge Biçiminde (PDF) çevrimiçi olarak yayınlanan elektronik materyal gerçek yayın kabul edilecektir. 2- Yeni takson isimleri için aranan Latince betim ya da diyagnoz gerekliliği, Latince veya İngilizce yazılmış betim veya diyagnoz gerekliliği şeklinde değiştirilecektir. Bunlara ek olarak, 1 Ocak 2013’ten itibaren uygulanmak üzere, mantar olarak kabul edilen organizmaların yeni isimlerinin geçerli olarak basılabilmesi için, tanınmış (MycoBank gibi) bir veri bankası tarafından verilen bir tanımlayıcının (seri numarasının) protologta (bir ismin geçerli basımında onunla ilgili verilen her türlü bilgi) belirtilmesi gerekmektedir. Elektronik yayın ile ilgili yeni maddelerin taslak metinleri ve bunların en iyi şekilde nasıl uygulanacağı aşağıda sunulmuştur.

*Uluslararası Alg, Mantar ve Bitki Adlandırma Yasası*’nda yapılan bu değişikliklerin geniş kitlelere duyurulmasını sağlamak amacıyla bu makale BMC Evolutionary Biology, Botanical Journal of the Linnean Society, Brittonia, Cladistics, MycoKeys, Mycotaxon, New Phytologist, North American Fungi, Novon, Opuscula Philolichenum, PhytoKeys, Phytoneuron, Phytotaxa, Plant Diversity and Resources, Systematic Botany ve Taxon dergilerinde yayınlanacaktır.

## Giriş

Avustralya’nın Melborn şehrinde 2011 yılının Temmuz ayında gerçekleştirilen XVIII. Uluslararası Botanik Kongresi’nde, yeni adı *Uluslararası Alg, Mantar ve Bitki Adlandırma Yasası* olan *Uluslararası Botanik Adlandırma Yasası*’nda 1 Ocak 2012 tarihinden itibaren yürürlüğe girecek iki önemli değişiklik yapıldı. Söz konusu değişiklikler bu *Yasa* uyarınca isim yayınlayan herkesi etkileyecektir. *Melborn Yasası*, 2012 yılının ortalarına kadar yayınlanamayacağından, özellikle elektronik yayıncılıkta gerçek yayın (Madde 29, 30 ve 31) konusu ile ilgili değişiklikleri ana hatlarıyla burada vermenin yararlı olacağını düşündük. Melborn’da kabul edilen *Yasa* ile ilgili tüm değişikliklerin özet raporu için McNeill ve ark. (2011)’na bakınız.

Editör ve yayıncıların *Yasa*’daki bu değişiklikleri en iyi şekilde uygulayabilmelerine yardımcı olmak amacıyla, gerçek yayın konusuyla ilgili Madde, Not ve Öneri-lerin değiştirilmiş taslak metinleri hazırlandı. Ayrıca, elektronik ortamda yeni isim yayınlamak ve tiplendirme yapmak isteyenler için, bu değişikliklerin ne anlama *geldiğini* veya *gelmediğini* ana hatlarıyla ortaya koyduk. Okuyuculara, kongre öncesinde önerilen değişikliklerin (Chapman ve diğ. 2010) yer aldığı Elektronik Yayın Özel Komitesi’nin raporunu da dikkate almalarını öneriyoruz. Çünkü o raporda *Melborn Yasası*’nda kabul edilen değişikliklerin nedenleri verilmektedir.

## Madde 29, 30, 31 ve Öneri 29A, 30A ile 31A’nın yeniden düzenlenen taslak metinleri

Aşağıda, ilgili tüm Madde, Not ve Önerilerin (Örnekler olmaksızın) yeni metinleri, değişiklikler **koyu** yazılarak verilmiştir. *Melborn Yasası*’nın yazılı baskısının son şekli Aralık 2011’deki Editörler Komitesi toplantısında oluşturulacağından, bu metin geçicidir..

### Madde 29

*29.1.* Bu Yasa’ya göre yayın, bütün halka ya da en azından botanikçilerin kullanabildiği kütüphanesi olan botanik enstitülerine (satış, değişim veya hediye yoluyla) dağıtılan basılmış metin ile gerçekleştirilir. **Yayın, ayrıca Uluslararası Standart Seri Numarası (ISSN) ya da Uluslararası Standart Kitap Numarası (ISBN)’na sahip Taşınabilir Belge Biçimindeki (PDF; ayrıca bkz. Madde 29.3 ve Öneri 29A.1) materyalin, çevrimiçi yayında elektronik olarak dağıtılmasıyla da gerçekleştirilebilir.** Yeni isimlerin genel bir toplantıda sunulması, halka açık bahçe veya koleksiyonlarda sergilenmesi, taslak metinden hazırlanmış mikrofilm, daktilo yazımları, herhangi bir şekilde hazırlanmış ama basılmamış materyal ya da **yukarıda tanımlanmış elektronik dağıtımdan başka bir şekilde yapılan** elektronik dağıtımla yayın gerçekleştirilmez.

***29.2.* Bu maddede yer alan “çevrimiçi” kelimesi, Dünya Ölçeğinde Ağ (www) üzerinden elektronik olarak erişilebilir olmayı ifade eder.**

***29.3.* Eğer Taşınabilir Belge Biçimi (PDF)’nin yerine bir başkası başarılı bir şekilde yerleşirse, o zaman Genel Komite (bkz. Bölüm III) tarafından uygun bulunacak bu biçim uluslararası standart biçimi olarak kabul edilebilir.**

***29.4.* Bir elektronik yayın çıktıktan sonra, bu yayında değişiklik yapılmamalıdır. Yapılacak herhangi bir değişiklik, kendi başına gerçek olarak yayınlanmış olmaz. Düzeltme ya da düzenlemelerin ayrı olarak gerçek bir yayın olarak çıkması gerekir.**

### Öneri 29A

[*Viyana Yasası*’ndaki ilgili Öneriler, aşağıdaki gibi değiştirilmiştir:]

***29A.1.* Taşınabilir Belge Biçiminde (PDF) elektronik olarak yapılan yayın, PDF/A arşivleme standartlarıyla uyumlu olmalıdır (ISO 19005).**

***29A.2.* Yazarlar arşivlenen ve olabildiğince kullanışlı olan aşağıdaki ölçütlere uygun yerlerde yayın yapmalıdırlar (ayrıca bkz. Öneri 29A.1)**:

**(*a*) Materyal, birden fazla güvenilir (örneğin ISO-sertifikalı) çevrimiçi dijital veri bankasına konuyor olmalıdır**;

**(*b*) Dijital veri bankaları dünyanın farklı yerlerinde, mümkünse farklı kıtalarında olmalıdır**;

**(*c*) Ayrıca dünyanın farklı yerlerindeki, tercihen farklı kıtalardaki kütüphanelerde basılı kopyalarının depolanıyor olması önerilir.**

### Madde 30

***30.1.* 1 Ocak 2012 tarihinden önce materyalin elektronik ortamda dağıtılmasıyla yapılan yayınlar gerçek yayın olmaz.**

***30.2.* Bir elektronik yayın, onun sadece geçici bir yayın olduğu veya olacağı, yayıncı tarafından son hali olarak karar verilen bir metinle değiştirileceğine dair yayının içinde ya da yayınla beraber verilen bir kanıt olduğunda, gerçek olarak basılmış olmaz. Bu durumda yayının yalnızca son hali gerçek olarak basılmıştır.**

*30.3.* Silinmez el yazısı ile 1 Ocak 1953’ten önce yapılan yayınlar gerçek yayındır. Sonraki tarihte hazırlanan silinmez el yazısı yayınlar gerçek olarak basılmamıştır.

*30.4.* Bu Madde gereğince, silinmez el yazısı materyal, bazı mekanik veya grafik işlemleri (örneğin taş baskı, ofset baskı veya asitle metal üzerine yazma metodu) sonucu çoğaltılmış olan el yazısı materyaldir.

*30.5.* Ticari kataloglarda veya bilimsel olmayan gazetelerdeki 1 Ocak 1953 ve sonrası ile tohum değişim listelerindeki 1 Ocak 1973 ve sonrası yayınlar gerçek yayın sayılmazlar.

*30.6.* Kurutulmuş materyal (exsiccatae) ile birlikte basılı materyalin 1 Ocak 1953 ve sonrasında dağıtımı, gerçek yayın oluşumunu sağlamaz.

*Not 1*. Eğer basılı metin ayrıca kuru materyalden bağımsız olarak ta dağıtılırsa, gerçek yayın olarak basılmış olur.

*30.7.* 1 Ocak 1953 ve sonrasında, bir ünvan almak amacıyla bir üniversite veya bir eğitim enstitüsüne tez olarak sunulmuş bağımsız ve düzenli aralıklarla yayınlanmayan bir yayın, yazarı ya da yayınlayıcısı tarafından onun gerçek bir yayın olarak kabul edildiğinin açık bir ifadesi (*Yasa*’nın gerçek bir yayın için gerekli koşullarına işaret eden) veya diğer içsel bir kanıt olmadıkça gerçek bir yayın olarak basılmış değildir.

*Not 2.* Orijinal baskıda Uluslararası Standart Kitap Numarası (ISBN)’nın veya basımevi, yayıncı veya dağıtımcı isimlerinin varlığı, bu yayının gerçek bir yayın olması amaçlı bir çalışma olduğunun içsel bir kanıtı olarak değerlendirilir.

### Öneri 30A

***30A.1.* Aynı elektronik yayının geçici ve son halinin gerçekte ne zaman ilk olarak yayınlandığının açıkça belirtilmesi gerekir.**

30A.2. Yazarların yeni isimleri ve yeni taksonların (adlandırma yenilikleri) betim veya diyagnozlarını ömrü kısa olan yayınların her çeşidinde, özellikle de metnin sürekliliğinin sınırlanabileceği ve bu nedenle de gerçek bir yayın için kopya sayısının belirgin olmadığı veya halka ulaşmasının mümkün olmadığı çok az ve belirsiz sayıda çoğaltılarak basılan materyallerde yayınlamamaları ısrarla önerilir. Ayrıca yazarların yeni isim, betim veya diyagnozları popüler dergilerde, özet dergilerinde veya düzeltme sayfalarında yayınlamaktan sakınmaları gerekir.

*30A.3.* Her yerde ve her zaman bulunabilir olmasına yardımcı olmak amacıyla, adlandırma yenilikleri yayınlayan yazarlar, düzenli olarak taksonomik makale yayınlayan dergileri tercih etmelidir. Eğer yayın yaptığı yer böyle bir dergi değilse, **yayının bir kopyası (basılı ya da elektronik materyal) söz konusu taksonomik grubun kayıtlarını tutan bir merkeze gönderilmelidir. Sadece basılı materyal olarak varlığını devam ettiren yayınların** dünyanın farklı yerlerindeki en az on, tercihen daha fazla sayıda botanik ya da genel kullanıma açık diğer kütüphaneler-de tutulması gerekir.

*30A.4.* Yazarlar ve editörlerin özette adlandırma yeniliklerinden bahsetmeleri veya bu yenilikleri yayında bir indekste listelemeleri tavsiye edilir.

### Madde 31

*31.1.* Bir gerçek yayının tarihi Madde 29 ve 30’da tanımlandığı gibi basılmış **ya da elektronik** metinin kullanılabilir olduğu tarihtir. Başka bir tarihin kabul edilmesi için kanıt olmadığında, basılı **ya da elektronik** metinde görünen tarih doğru olarak kabul edilmelidir.

[*Viyana Yasası*’ndaki Not 1, aşağıdaki gibi değiştirilmiştir:]

***31.2.* Yayının elektronik ve basılı halleri birlikte gerçekleştirildiğinde, bu iki ayrı yayın şeklinin tarihleri Madde 31.1’e göre farklı olmadığı sürece, aynı tarihte gerçek olarak basılmış kabul edilirler.**

*31.3.* Satışa konan süreli yayın veya diğer çalışmaların ayrı sayıları önceden yayınlanırsa, bu parça üzerindeki tarih, bunun yanlışlıkla yapıldığına ilişkin bir kanıt olmadıkça, gerçek yayın tarihi olarak kabul edilir.

### Öneri 31A

*31A.1.* Yayıncı veya ilgili dağıtım şirketinin basılmış metni, dağıtmak üzere normal taşıyıcılara teslim ettiği tarih, onun gerçek yayın tarihi olarak kabul edilmelidir.

## En iyi şekilde uygulanması

Yeni isim içeren yayınların *Melborn Yasası*’na uygun olması için, tüm yeni isim yazarlarının, editörlerin ve yayıncıların gerekli ilgiyi göstermesi gerekir. Böylece yayınlanan isimler gerçek bir yayında basılmış olacaktır. Çevrimiçi baskıları olan dergi, monograf serisi ve kitaplarda yayın yapacak olanlara, bu kuralların toplumda hızla yerleşmesinde etkili olacağı düşüncesiyle, editörlerle iletişim halinde olmalarını öneriyoruz. Birçok yayıncı bilimsel yeniliklerin olduğu e-yayını basarken dikkatli davranmaktadır (bkz. Knapp and Wright 2010; PLoSOne [http://www.plosone.org/static/policies.action#taxon] yönergesi). Önemli uygulamaları içeren *Yasa*daki bu değişikliklerin yapılmasına olan gereksinim zaman içinde açık bir şekilde ortaya çıkmıştır.

*Melborn Yasası*’na uygun adlandırma yeniliği içeren e-yayınların başlangıç aşamasında, faydalı olacağını düşündüğümüz bazı uygulamalar aşağıda sunulmuştur:

• Her makalenin yayın tarihini (*New Phytologist* ya da *Nature* dergisi gibi çok sayıdaki dergide olduğu gibi) belirgin bir biçimde üzerinde taşıması gerekir.

• Eğer erken çıkan çevrimiçi baskı son baskısından farklı ise (bu haliyle gerçek yayın değildir) bu durumun belirgin biçimde her makalede belirtilmesi gerekir (örn. *American Journal of Botany*).

• Her makalede ilgili yayının ISSN ya da ISBN numaralarının göze çarpar şekilde belirgin basılması, gerçek yayınların kataloğunu hazırlayanlara yardımcı olacaktır.

• CLOCKSS sisteminde (betim için bkz. Knapp and Wright 2010) ya da diğer uluslararası arşivleme ve koruma sistemlerinde yer alan dergilerde (ya da monograf serilerinde) yapılan yayın, uzun süreli arşivlemeyi güvenceye alacaktır.

• Öneri 30A.3.’de tavsiye edildiği gibi, elektronik olarak yayın yapan yeni isim yazarlarının uygun kayıt merkezlerini bilgilendirmeleri gerekir; bu durum elektronik olarak yayınlanan ismin farkında olmayan kayıt personeline yardımcı olacaktır.

## Bu değişiklikler hangi anlama *gelmez*

*Yasa*’daki yeni Madde ve Önerilerde PDF ve PDF/A terimleri kullanılsa da, bu yayınların geçerli olabilmesi için *sadece* bu biçimde yayınlanması zorunludur anlamına gelmez. Örneğin, bazı çevrimiçi dergiler makaleleri PDF biçimiyle birlikte HTML (Bağlantılı Metin İşaretleme Dili) biçiminde de yayınlamaktadırlar. Bu gibi durumlarda PDF biçimindeki baskı gerçek yayın olarak yayınlanmıştır. Taşınabilir Belge Biçiminden daha başarılı bir başka biçimin olması durumunda, Genel Komitenin uygun bulacağı yeni bir uluslararası standart biçimini kabul edebilecek olması, yeni isim yazarlarının ve bu *Yasa*’yı uygulayan toplumun yeni ilerlemeler karşısında gelişmelerden haberdar edileceği ve *Yasa*’nın çağın gerisinde bırakılmayacağı anlamına gelir.

• Aşağıda belirtilen şekillerde yapılan elektronik adlandırma yayınları, *Melborn Yasası* uyarınca, gerçek yayın olarak *sayılmazlar*.

• Yayının ağ sitelerinde (bilgisunar-website) ya da internet aracılığıyla ulaşılabilen kısa süreli metinlerde yer alması (ISSN’nin alınabilmesi için sıkı kurallar bulunmaktadır [http://www.issn.org/]).

• Yayının ISSN ya da e-ISNN’e kayıtlı olmayan dergilerde yer alması.

• Yayının ISBN yada e-ISBN’e kayıtlı olmayan kitaplarda yer alması.

Onaylanan yeni Öneri, e-yayına ait basılı bir kopyanın (ayrı baskının) botanikçilerin kullandığı bir kütüphanede bulunmasını tavsiye etmektedir, ancak bu konuda kütüphanecilerin uyması gereken standart bir uygulama ya da protokol getirmez. Kütüphanecilerin kendileri de farklı yayın yöntemleri arasında karmaşık geçiş kuşağında yer almaktadır (Johnson and Luther 2007). Botanikçiler, kütüphanecileri belli bir büyüklüğe sahip eserlerin saklanmasında başarılı, ancak ayrı tek baskıları saklama konusunda isteksiz ya da başarısız bulabilirler.

## Isim yayınlamaya ilişkin *Yasa*’da yer alan diğer iki önemli değişiklik

Melborn’da kabul edilen ve 1 Ocak 2012 tarihinden itibaren geçerli olacak olan *Yasa*’daki ikinci değişiklik, bu *Yasa* kapsamında değerlendirilen tüm organizmaların yeni takson isimlerinin geçerli yayınlanabilmesi için gerekli betim veya diyagnozun İngilizce ya da Latince olabileceğidir. Bu koşul bitki fosilleri için şu anda uygulanmaktadır, ancak fosil olmayan tüm yeni taksonlar için betim veya diyagnozun Latince olması gerekmektedir (mantar ve bitkiler için 1 Ocak 1935’den beri; algler [eğer bu *Yasa* kapsamında değerlendirilirse siyanobakterler dahil] için 1 Ocak 1958’den beri). Bu değişikliklerin bilimsel isimlerin oluşturulmasına bir etkisi yoktur, bundan sonra da isimler Latince olmaya ve Latince dilbilgisi yapısına göre oluşturulmaya devam edecektir. Betim ve diyagnozların sadece Latince veya İngilizce olması ya da ikisinin de kabul edilmesi elbette dergilerin editörleri tarafından tercih edilecektir.

Melborn’da kabul edilen ve 1 Ocak 2013’ten sonra (Miller ve ark. 2011’de belirtildiği üzere 1 Ocak 2012 tarihinde değil) geçerli olacak *Yasa*da isimlerin yayınlanmasına ilişkin yapılan üçüncü değişiklik, mantar olarak kabul edilen organizmaların yeni isimlerinin geçerli olarak basılabilmesi için, tanınmış (MycoBank gibi [http://www.mycobank.org/]) bir veri bankası tarafından verilen bir tanımlayıcının (seri numarasının) protologta (bir ismin geçerli basımında onunla ilgili verilen her türlü bilgi) belirtilmesi-nin gerekliliğidir. Yeni mantar isimleri için kayıt yaptırma gereklilikleri başka bir yerde ayrıca yayınlanacaktır.

Yeni mantar isimleri için 1 Ocak 2013 ve sonrasında geçerli olacak belli bir tanımlayıcıya olan gereklilik, bitki ya da alglere *uygulanmayacaktır*. Bu gruplardaki yeni isim yazarlarının kayıt merkezlerinden Yaşam Bilimi Tanıtıcı İsmi (LSID) ya da başka bir tanıtıcı isim almasına gerek yoktur.
